# Influence of Deformation Degree on Microstructural Evolution and Tensile Behavior of TiB-Reinforced IMI834 Composites

**DOI:** 10.3390/ma18102306

**Published:** 2025-05-15

**Authors:** Baobing Wang, Mingliang Liu, Zhiwei Zhao, Jiuxiao Li, Minhao Fan, Ziyi Li

**Affiliations:** 1 State Key Laboratory of Metal Matrix Composites, School of Materials Science and Engineering, Shanghai Jiao Tong University, Shanghai 200240, China; 2 School of Materials Science and Engineering, Shanghai University of Engineering Science, Shanghai 201620, China

**Keywords:** TiB whiskers, IMI834 titanium alloy, hot rolling, texture evolution, Abaqus simulations, tensile properties

## Abstract

Modern aero-engines need alloys that sustain both strength and ductility at high temperatures. However, conventional titanium alloys face inherent trade-offs between strength and ductility. In situ TiB-reinforced titanium matrix composites could fill this gap, but their texture evolution and hot-working mechanics are still poorly understood. In this study, TiB-reinforced IMI834 titanium matrix composites were synthesized using in situ technology in a remelting furnace. Meanwhile, the evolution of microstructure and texture in the hot-rolled titanium matrix composites was examined through both Abaqus simulations and experimental observations. Results indicate that dynamic recrystallization occurred in the microstructure of the composites at a deformation level of 95%. Due to the specific orientation relationship between the TiB whiskers and Ti matrix, the hot-rolled composites developed a pronounced [11-20]_Ti_ // rolling direction fiber texture. TiB whiskers rotated toward the rolling direction, enhancing the intensity of the [11-20]_Ti_ // rolling direction fiber texture, consistent with the predictions from numerical simulations. Tensile tests revealed that the combined effects of grain refinement and the rotation of TiB whiskers along the rolling direction increased the yield strength of the hot-rolled composite to 1153 MPa, while simultaneously raising the elongation to 10%.

## 1. Introduction

Titanium matrix composites (TMCs) have garnered substantial attention across various industries, including aerospace, defense, and automotive sectors due to their exceptional specific strength, excellent corrosion resistance, and superior temperature performance [1,2,3,4,5,6]. Among the various fabrication techniques employed for TMC production, in situ reaction synthesis technology is widely adopted because of its low production cost and minimal interfacial contamination [7,8]. A variety of reinforcements, such as TiB, TiC, Al_2_O_3_, SiC, and TiB_2_ have been incorporated into TMCs, with TiB standing out due to its excellent thermodynamic stability, strong interfacial bonding with the matrix, and high chemical compatibility [9,10]. These properties enable efficient load transfer, grain refinement, and suppression of grain growth, contributing significantly to the mechanical performance of TMCs [3,10,11,12].

Research on TiB-reinforced composites focused mostly on α or α + β titanium alloy matrices like Ti–6Al–4V. When reinforced with TiB, these alloys promise even greater strength levels. Meanwhile, thermo-mechanical processing (TMP) techniques such as hot forging, rolling, and extrusion are critical for controlling the microstructure of the materials and, thus, optimizing their properties [5,13,14]. Huang et al. [15,16] have underscored that the mechanical performance of TMCs is governed by the interaction between reinforcements and the matrix, as well as grain refinement driven primarily by dynamic recrystallization during TMP. TMP can break up and redistribute brittle as-cast TiB networks, refine matrix grains via dynamic recrystallization, and homogenize the reinforcement distribution. For example, TiB tends to form continuous columnar networks along grain boundaries in as-cast microstructures [17]. Thermal processing at sufficient strain (high extrusion ratios or reduced heavy forging) eliminated porosity and produced a fine microstructure [18].

In addition to producing a more uniform and finer-grain organization, another key outcome of hot working TiB/Ti composites is the pronounced orientation of the reinforcement, which has a strong influence on mechanical behavior. During processes like extrusion or rolling, TiB whiskers become strongly aligned along the principal working direction [14]. The degree of alignment generally increases with the amount of deformation imposed [19]. This oriented microstructure is beneficial for tensile loading along the alignment direction [20,21]. Wang et al. [22] explored the composite reinforced with 3.5 vol.% TiB whiskers exhibited optimal mechanical performance, achieving a tensile strength of 1248 MPa and an elongation of 10.5% at room temperature. This structural reorientation, together with TMP-induced grain refinement, directly improves the strength and mechanical performance. However, the evolution of reinforcement orientation and matrix texture during TMP introduces significant anisotropy in mechanical behavior. Studies have shown that aligned TiB whiskers and matrix crystallographic textures synergistically affect strength and ductility along different loading directions [23]. Le et al. [19] demonstrated that a strong matrix texture combined with aligned TiB whiskers led to higher strength, but lower ductility, when loading perpendicular to the whisker alignment, compared to loading parallel. Furthermore, Ma et al. [24] also found that TiB whiskers can induce dynamic recrystallization and inhibit grain coarsening, promoting a stable microstructure. Despite the enhancement in strength, achieving a favorable strength–ductility balance remains challenging, as increased TiB content or whisker misalignment may lead to reduced toughness.

Despite these advances, systematic studies focusing on hot-rolled TMCs remain limited, particularly regarding the influence of whisker or short-fiber orientation on mechanical behavior. Therefore, it is important to further investigate the effect of reinforcement on the mechanical properties of TMCs. In this study, TiB/IMI834 composites were fabricated via in situ synthesis, and the influence of deformation on their microstructure, texture, and mechanical properties was systematically investigated. Special attention was given to the orientation behavior of TiB whiskers under different rolling reductions, supported by numerical simulations using Abaqus. This work aims to provide further insight into the microstructural evolution and mechanical response of TiB/IMI834 composites processed by the hot rolling process technology.

## 2. Materials and Methods

### 2.1. Numerical Simulation Method

**Figure 1:**
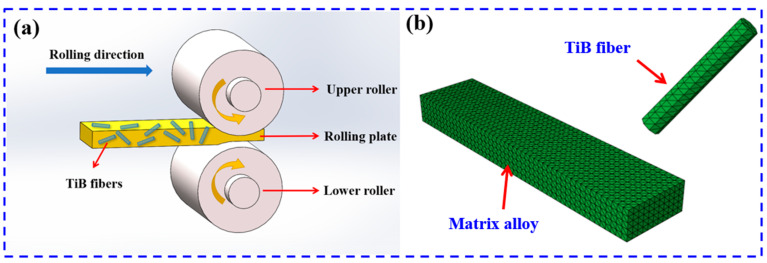
(**a**) The schematic diagram of the hot rolling process; (**b**) finite element meshing.

Three-dimensional modeling was performed through Deform-3D v10 software, followed by software simulation through Abaqus. Figure 1a shows the 3D model of the equal-axis double-roll rolling bar. The plate model was meshed by tetrahedra and imported into the Abaqus simulation environment, as illustrated in Figure 1b. Material constitutive models for the matrix alloy and TiB whiskers were established, and boundary condition parameters were set. The deformation process was simulated at a strain rate of 0.01 s^−1^ and a temperature of 1010 °C. To represent the random distribution of whiskers, 60° was chosen as a representative mean to define the initial conditions in the numerical model [25]. The plate was rolled in the same direction until a total thickness reduction of 95% was achieved. The numerical simulation results were observed at the post-processing interface.

### 2.2. Experimental Procedure

**Figure 2:**
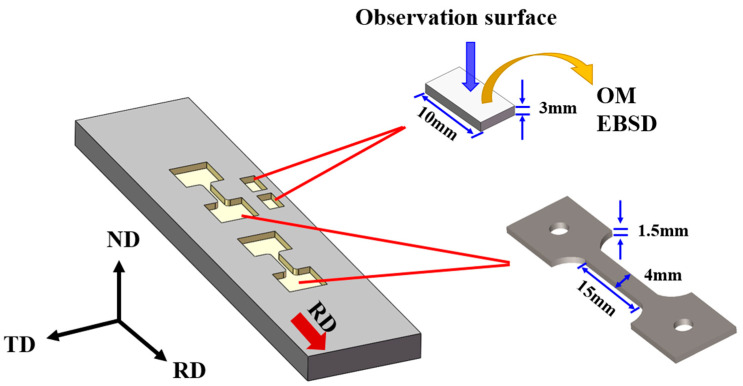
The positions for microstructure observation and tensile tests. (RD, ND, and TD stand for the rolling direction, normal direction, and transverse direction, respectively).

TiB-reinforced TMCs were fabricated by in situ synthesis technology using IMI834 titanium alloy as the matrix, and the theoretical volume fractions of TiB whiskers were 1.82%. The plate is rolled at 1010 °C until the total deformation is 95% by an equal-axis double-roller. For microstructural and tensile properties analysis, the test samples were sectioned from the rolled plates using wire electrical discharge machining, and dog-bone tensile specimens (gauge dimensions 15 mm × 4 mm × 1.5 mm) were machined from both the as-cast and rolled plates with their tensile axes parallel to the rolling direction (RD), as shown in Figure 2. The evolution of microstructure and crystallographic texture in the hot-rolled TMCs was characterized using an optical microscope (OM, Japan Keyence VHX-5000,KEYENCE Corporation, Osaka, Japan) and a scanning electron microscope (SEM, Germany Zeiss Gemini 300, Carl Zeiss AG, Jena, Germany) equipped with electron backscattering diffraction (EBSD, Symmetry S3, Oxford Instruments NanoAnalysis, High Wycombe, UK). OM specimens were prepared by standard metallographic grinding and polishing, followed by etching with Kroll’s solution (HF:HNO_3_:H_2_O = 1:3:10). EBSD scans were performed with a step size of 0.4 μm over a 300 × 300 μm^2^ area, and the data were post-processed using OIM analysis 7 software. Uniaxial tensile tests were conducted at room temperature at an initial strain rate of 10^−3^ s^−1^ using a universal testing machine (Zwick Z50, ZwickRoell GmbH & Co. KG, Ulm, Germany).

## 3. Results and Discussion

### 3.1. Numerical Simulation Results

**Figure 3:**
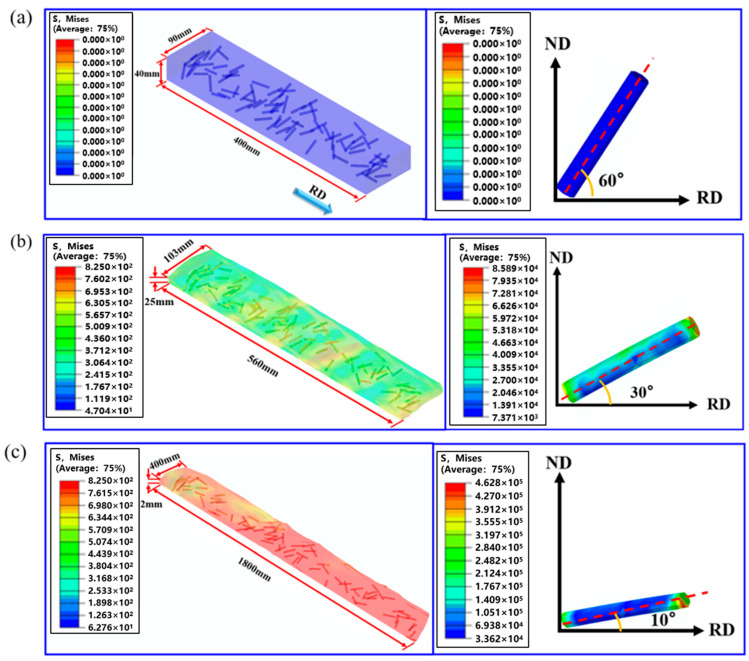
Crystal-plasticity finite-element (CPFE) predictions of TiB-reinforced IMI834 plate during hot rolling: (**a**) initial state, TiB whisker inclined 60° to the RD; (**b**) 40% reduction; (**c**) 95% reduction.

Figure 3 shows the crystal-plasticity finite-element (CPFE) predictions of the stress distribution in the TiB-reinforced IMI834 plate during hot rolling. The initial state of the composite plate before rolling assumes a TiB whisker orientation angle of 60° relative to the RD, as shown in Figure 3a. At a rolling reduction of 40% (Figure 3b), elevated stresses appear at both whisker tips, creating a torque that rotates the whisker toward the RD and lowers its inclination to approximately 30°. When the total reduction reaches 95% (Figure 3c), the stress concentration intensifies, causing a greater degree of whisker alignment along the RD. At this stage, the whiskers are nearly parallel to the RD, with an orientation angle reduced to approximately 10°. These simulation results demonstrate that increasing rolling reduction leads to progressive TiB whisker rotation toward the RD, primarily driven by the rise in flow stress during hot deformation.

**Figure 4:**
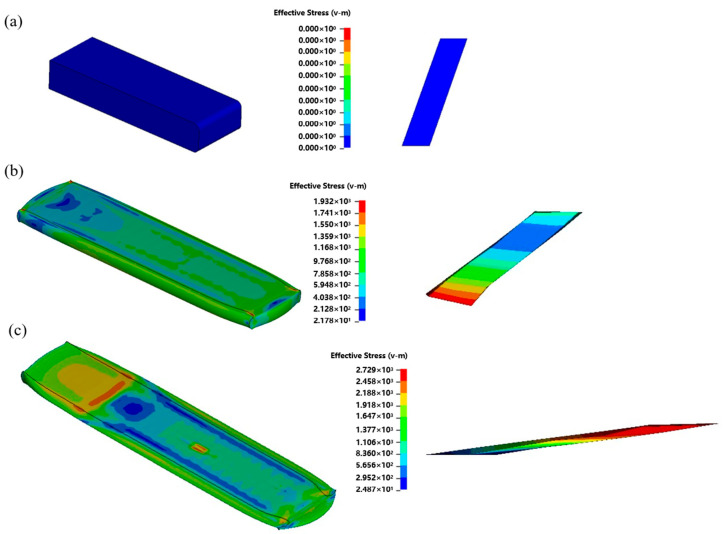
Predictions of the stress distribution in TiB-reinforced IMI834 plate during hot rolling: (**a**) initial state; (**b**) 40% reduction; (**c**) 95% reduction.

Figure 4 illustrates the effective stress distribution in the TiB-reinforced IMI834 composite during the hot rolling process, as predicted by finite element simulation. The simulation reveals how stress evolves with increasing rolling reduction. Before deformation (Figure 4a), the plate shows negligible internal stress, as expected for the undeformed billet. The stress remains close to zero throughout the matrix, indicating a stress-free initial condition. When the plate undergoes a deformation with a thickness reduction of 40% (Figure 4b), noticeable stress concentrations begin to develop, particularly at the whisker–matrix interfaces. Regions near the edges and corners of the plate experience elevated stress levels due to geometric constraints. The matrix displays the stress gradient from the center to the surface, where the whiskers start to rotate along the RD under torque. Figure 4c shows that 95% of the severe plastic deformation leads to a significant increase in the effective stress, particularly near the surface layers and the regions containing aligned TiB whiskers. The plate exhibits distinct strain localization zones, and the matrix material around the whiskers is highly stressed. The deformation-induced rotation of whiskers toward the RD reaches its maximum extent at this stage, consistent with the orientation change.

These results emphasize that the mechanical interaction between the matrix and the TiB whiskers under compressive strain leads to stress concentration zones which, in turn, promote whisker realignment along the rolling direction. This evolution of stress and orientation enhances the composite’s anisotropic behavior and reflects the strong coupling between plastic deformation and reinforcement during hot rolling.

### 3.2. Experimental Results

**Figure 5:**
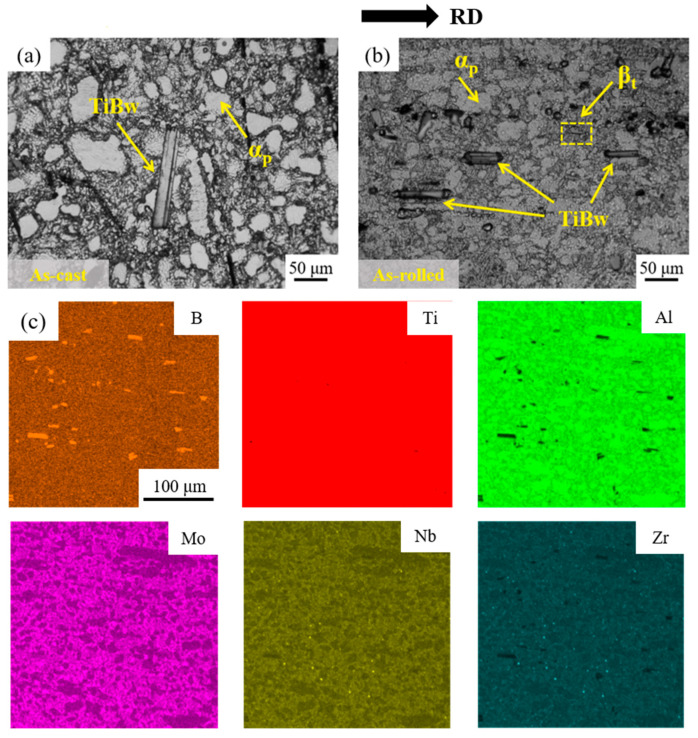
The microstructure of TiB/Ti composites in different processing states: (**a**) as-cast; (**b**) hot-rolled; and (**c**) the elemental distribution of the hot-rolled state.

Figure 5 shows the microstructure of TiB/Ti composites at different processing states. The as-cast composites exhibit a typical bimodal microstructure, primarily composed of equiaxed primary *α* phase (*α_p_*), with TiB whiskers (TiBw) randomly dispersed throughout the matrix, as shown in Figure 5a. The hot-rolled composite with 95% reduction displays a refined microstructure composed of recrystallized equiaxed grains (*α_p_*) and a small fraction of lamellar *β* transformation (*β_t_*), as depicted in Figure 5b. Figure 5c shows the EDS elemental mapping for the hot-rolled state. The map for Ti shows a uniform spread throughout the material, and the elemental map for B shows the distribution of TiB whiskers within the matrix. Al, Mo, Nb, and Zr elements represent alloying elements in the Ti matrix. The distribution of these elements helps to identify the solid solution strengthening and phase formation.

Hot rolling induces both grain deformation and recrystallization in titanium matrix composites. Previous studies [26,27] have reported that plastic deformation plays a major role and the recrystallization behavior is inhibited with small deformation (≤80%), whereas higher deformation promotes recrystallization. In this study, the rolling deformation reached 95%, allowing for complete recrystallization. Therefore, fine equiaxed α grains are the main microstructure of titanium matrix composites. Meanwhile, TiBw are observed to be well aligned along the RD and exhibit strong interfacial bonding with the matrix. In addition, most of the TiBw remain intact after rolling, owing to their high modulus, elevated melting point, and superior strength [11,12,28]. These observations are consistent with the numerical simulation results.

**Figure 6:**
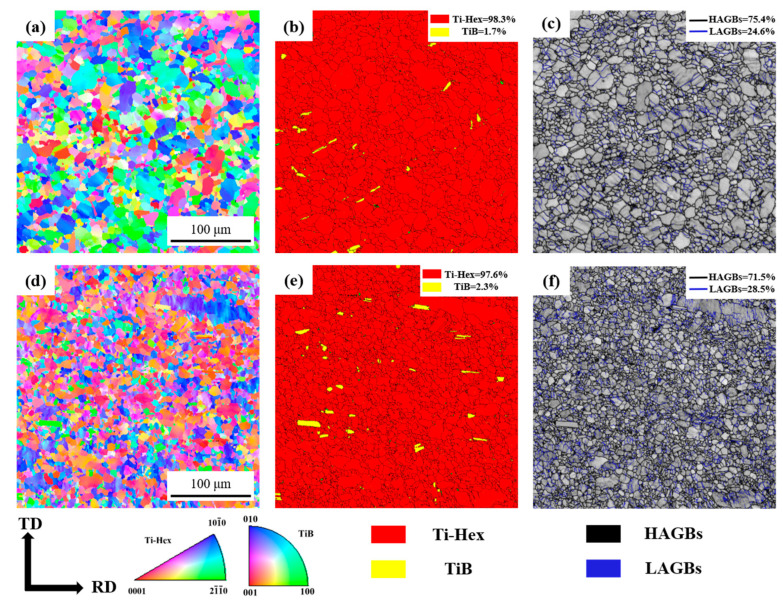
EBSD inverse pole figure maps, phase distribution, and grain boundaries: (**a**–**c**) as-cast and (**d**–**f**) as-rolled composites.

**Figure 7:**
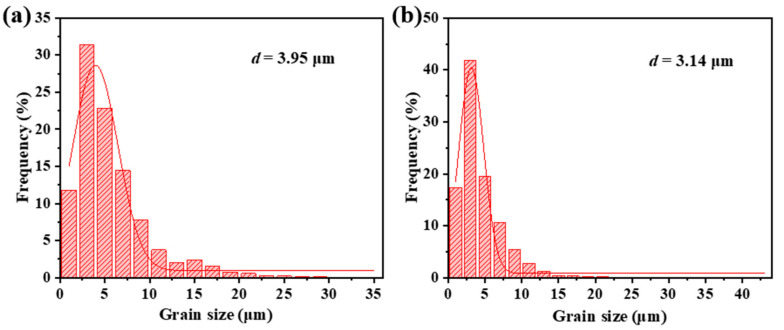
Grain size distribution of: (**a**) as-cast and (**b**) as-rolled composites.

To investigate the microstructure characteristics of different phases during the rolling deformation, the EBSD figures of the as-cast and as-rolled composites are presented in Figure 6. Figure 6a shows the inverse pole figure (IPF) map of the as-cast composite. It can be observed that the grain distribution of the three characteristic crystal directions of Ti-Hex is relatively uniform, and there is no obvious preferred orientation. Combined with Figure 7a, the matrix microstructure of the as-cast composite is equiaxed crystalline with an average size of about 3.95 μm. Figure 6b shows the distribution of Ti-hexagonal (Ti-Hex) and TiB phases, where Ti-Hex constitutes 98.3% of the material, and TiB is dispersed in the matrix, accounting for about 1.7%. In addition, the distribution of TiBw is also about 60° from the RD, similar to the simulation angle. Figure 6c shows grain boundary map of the as-cast composites, the matrix microstructure mainly consists of a relatively high proportion (75.4%) of high angle grain boundaries (HAGBs, >15°) (marked in black), and accompanied by 24.6% low angle grain boundaries (LAGBs, 2–15°) (marked in blue).

Figure 6d shows the IPF map of as-rolled composites. The grain organization is significantly refined after hot rolling. A large number of dynamically recrystallized grains are present in the matrix and the vicinity of TiBw, with an average grain size of only 3.14 μm, as shown in Figure 7b. In addition, the orientation of TiBw after hot rolling is different from the as-cast state. Alignment of TiBw along the RD, as shown in Figure 6e, which is consistent with the results in the numerical simulation in Figure 3 and the OM images in Figure 5. Figure 6f shows the grain boundaries of the as-rolled composites. The proportion of LAGBs increased to 28.5% due to insufficient dynamic recovery during hot rolling, resulting in many small-angle sub-grain boundaries were formed. These changes are crucial for understanding the mechanical properties and performance of composites in the deformed state.

**Figure 8:**
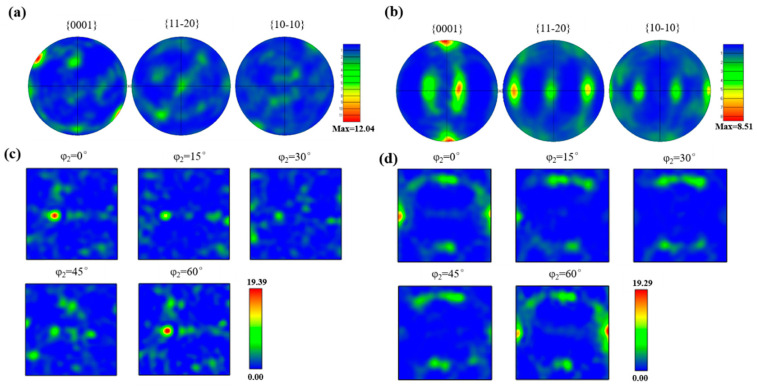
{0001}, {11-20} and {10-10} pole figures and ODF: (**a**,**c**) as-cast; and (**b**,**d**) as-rolled conditions.

The evolution law of texture in titanium alloys has been widely studied by many researchers [29,30,31,32,33]. In the present work, the texture evolution of matrix alloy composites and the effect of TiBw on the texture evolution during rolling deformation were studied. The pole figures (PF) of {0001}, {11-20}, and {10-10} characteristic surfaces of α phase before and after hot rolling, as shown in Figure 8. Figure 8a shows the polar figures of the as-cast composites. The {0001} pole figure reveals that the texture is about 45° to the RD with a maximum multiple of uniform density of 12.04, and there is no obvious texture phenomenon on the {11-20} and {10-10} pole figures, where the grain orientation distribution is relatively uniform. The pole figures of the as-rolled composites as shown in Figure 8b. The α-phase grains form strong texture distribution characteristics on the {0001} plane. The polar density figures of the {0001} pole figure mainly distribute around TD with a maximum multiple of uniform density of 8.51, the texture of the {11-20} pole figure is mainly distributed on the rolling direction RD line. It can be concluded that the [11-20] crystal of the α-phase grain rotates to the RD by comprehensively analyzing the pole figures of the three characteristic crystal planes. It is reported that the composites of hot-rolled form a special texture due to the orientation relationship between TiBw and Ti matrix: [11-20]_Ti_ // [010]_TiB_, and TiB reinforcement grows in situ along its own [010] direction when the composites are prepared in situ [34]. In addition, the TiB whiskers primarily remain stable below 800 °C, and there is a tendency for coarsening of the TiB phase beyond 800 °C, which could influence the mechanical properties, particularly under tensile stress. Failure modes will range from nanometer-scale load-bearing fracture to micrometer-scale interfacial debonding [35].

Figure 8c,d show the orientation distribution function (ODF) for the α phase in both as-cast and as-rolled conditions. The ODF sections at φ_2_ = 0° and 60° exhibit pronounced texture components with peak orientation densities exceeding 19, exceeding other orientation densities across the Euler space. The concentration of orientations along these sections supports the dominance of a [11-20] // rolling direction alignment. This observation aligns with the pole figure analysis, and validates the presence of strong deformation-induced texture in the TiB-reinforced IMI834 matrix. Therefore, for the hot-rolled composites, the TiBw would gradually rotate to the RD. These results provide evidence to support that the TiB whisker rotation acts as a strain accommodation mechanism.

**Figure 9:**
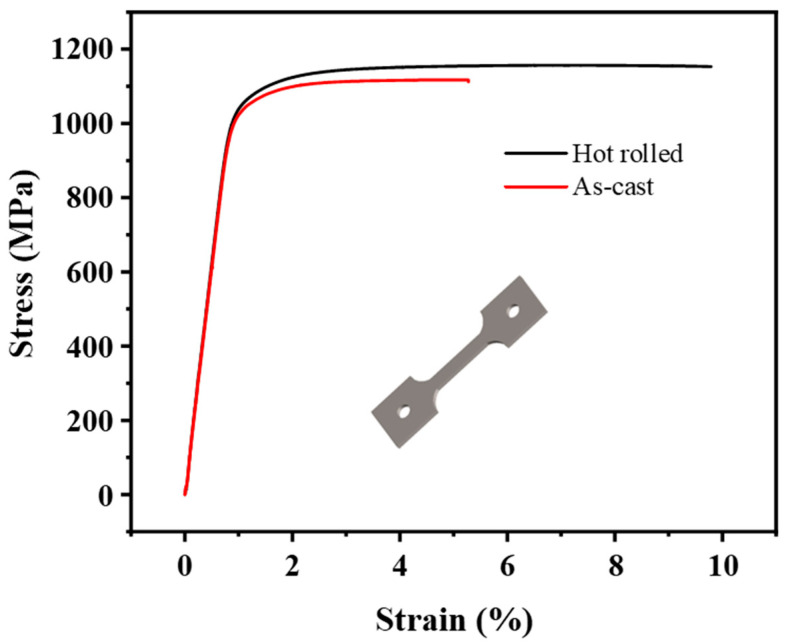
Room-temperature tensile engineering stress-engineering strain curves.

Figure 9 shows the room-temperature engineering stress–engineering strain curves of the as-cast and as-rolled composites. The results show that the yield strength of as-cast specimens is 1117 MPa, and the elongation is 5.28%. The main reason for the low elongation of the as-cast sample is that coarse grains cause fewer grain boundaries that hinder dislocation movement. Therefore, dislocation accumulation and winding are easy to occur at the interface between reinforcement and matrix, resulting in stress concentration and microcracking of the reinforcement during tension [36]. The yield strength of hot-rolled specimens reaches 1153 MPa, and the elongation is 10% significantly. The yield strength of the hot-rolled composites increased to a certain extent, and the plasticity increased significantly by 89%, which is due to the dynamic recrystallization of the matrix structure of the composites and the formation of fine grains during the hot rolling. According to the Hall–Petch formula [37],

(1) $$\sigma_{m}=\sigma_{o}+k_{m}d^{-1/2}$$

where *σ_m_* is the yield strength of materials, *σ*_0_ is a constant, *k_m_* is a constant of slope, and *d* is the average grain size. According to the above relationship, a smaller grain size enhances the yield strength due to the increased number of grain boundaries, which act as barriers to dislocation motion. In hot-rolled composites, where dynamic recrystallization leads to the formation of finer equiaxed grains, the uniform distribution of dislocations reduces the likelihood of crack initiation and propagation, especially in the direction of RD [38]. Similar findings were also reported in [39,40], the increased plasticity in the hot-rolled composites is linked to the reduced grain size and the corresponding decrease in dislocation pile-up.

In addition to grain size, the orientation development of the reinforcement in the matrix significantly affects the tensile properties. The reinforcement mechanism of short fiber reinforced TMCs is mainly based on the reinforcement bearing theory: the force acting on the matrix is transmitted to the reinforcement through the coordination of shear stress at the interface between the matrix and the reinforcement in the process of stress in service [31,41]. According to the strengthening formula of TiB/Ti composites [25],

(2) $$\sigma_{c}=\sigma_{m}\left[\frac{1}{2}\alpha \upsilon_{f}\left(\frac{l}{d}+2\right)+1-\upsilon_{f}\right]$$

where *σ_c_* is the strength of the composites, *σ_m_* is the strength of the matrix alloy, $\upsilon_{f}$ is the volume fraction of TiB whisker, *l*/*d* is the aspect ratio of TiB whisker, and *α* is the degree of dislocation of TiBw. The TiB whiskers in TMCs, typically elongated with an aspect ratio (*l*/*d*) greater than 1, exhibit a strong directional dependence of mechanical properties. When the tensile load is aligned with the direction of the whisker orientation, the composite exhibits improved strength and ductility compared to when the load is applied perpendicular to the whisker alignment, as discussed by Gorsse [42]. Furthermore, according to previous studies, the orientation of TiBw on the anisotropy of the mechanical properties in hot rolled TMCs is highly relevant, which is the synergistic effect of the matrix texture and the aligned TiBw [19,23].

In summary, the hot rolling process, which promotes the alignment of TiB whiskers and refines the matrix microstructure, significantly enhances the strength and ductility in the rolling direction. The reinforcement mechanism, driven by the effective load transfer from the matrix to the TiB whiskers, is a key factor in improving the material’s tensile properties.

## 4. Conclusions

In this study, the rotation behavior of TiBw during hot rolling was investigated through numerical simulation, while the microstructural evolution and tensile properties of TiB/Ti composites were examined experimentally. The texture evolution was characterized, and the correlation between mechanical properties and microstructural changes was analyzed. The main conclusions are as follows:

(1) Numerical simulation results revealed that TiBw progressively rotate toward the RD during hot rolling, becoming nearly parallel to the RD at a rolling reduction of 95%.
(2) EBSD analysis showed that the grain of the hot-rolled composites is refined and forms recrystallization texture and significant [11-20]_Ti_ // RD fiber texture. The rotation of TiBw to RD during hot rolling helps to improve the [11-20]_Ti_ // RD fiber texture due to the special orientation relationship between TiB and Ti matrix.
(3) Room-temperature tensile testing revealed that the as-cast composite exhibited a yield strength of 1117 MPa and an elongation of 5.28%. In contrast, the as-rolled composite demonstrated enhanced mechanical performance, with a yield strength of 1153 MPa and elongation of 10%. This improvement is attributed to a combined strengthening effect from grain refinement and the rotation of TiBw.

## Data Availability

Data will be made available on request. Further inquiries can be directed to the corresponding author.
